# Establishment of a highly sensitive sandwich ELISA for the N-terminal fragment of titin in urine

**DOI:** 10.1038/srep39375

**Published:** 2016-12-19

**Authors:** Nobuhiro Maruyama, Tsuyoshi Asai, Chiaki Abe, Akari Inada, Takeshi Kawauchi, Kazuya Miyashita, Masahiro Maeda, Masafumi Matsuo, Yo-ichi Nabeshima

**Affiliations:** 1 Diagnostic & Research Reagents Division, Immuno-biological Laboratories Co., Ltd. 1091-1 Naka, Fujioka-shi, Gunma 375-0005, Japan; 2 Department of Physical Therapy, Faculty of Rehabilitation, Kobe Gakuin University, 1-1-3 Minatojima-Minamimachi Chuo-ku, Kobe 650-0047, Japan; 3 Laboratory of Molecular Life Science, Institute of Biomedical Research and Innovation Foundation for Biomedical Research and Innovation, 2-2 Minatojima- Minamimachi Chuo-ku, Kobe 650-0047, Japan

## Abstract

Muscle damage and loss of muscle mass are triggered by immobilization, loss of appetite, dystrophies and chronic wasting diseases. In addition, physical exercise causes muscle damage. In damaged muscle, the N-terminal and C-terminal regions of titin, a giant sarcomere protein, are cleaved by calpain-3, and the resulting fragments are excreted into the urine via glomerular filtration. Therefore, we considered titin fragments as promising candidates for reliable and non-invasive biomarkers of muscle injury. Here, we established a sandwich ELISA that can measure the titin N-terminal fragment over a biologically relevant range of concentrations, including those in urine samples from older, non-ambulatory Duchenne muscular dystrophy patients and from healthy donors under everyday life conditions and after exercise. Our results indicate that the established ELISA could be a useful tool for the screening of muscular dystrophies and also for monitoring the progression of muscle disease, evaluating the efficacy of therapeutic approaches, and investigating exercise-related sarcomeric disruption and repair processes.

Titin (UniProtKB accession No. Q8WZ42)^1^,^2^, firstly called connectin^3^, is expressed in all striated muscles and is one of the largest known proteins, with a molecular weight in the range of 3.0 to 3.7 MDa. It spans a half-sarcomere from the Z-disc to the M-line, and it serves as a scaffold for sarcomerogenesis and myofibrillar assembly^4^. It has long been recognized that muscle damage and muscle fiber contraction initiate a cascade consisting of (1) cytoskeletal and sarcomeric disruption, including calpain-mediated degradation of titin^5^; (2) the inflammatory response of the damaged muscle^6^; and (3) a concomitant titin-related hypertrophic response that supports sarcomerogenesis and fiber repair^7^. Indeed, titin fragments were detected by mass spectrometric analysis in serums collected from young Duchenne muscular dystrophy (DMD) patients between the ages of 3 and 4 years old^8^, and comprehensive proteome studies of serum^9^ and urine^10^ of DMD patients and healthy donors revealed that titin showed the highest fold-change between healthy subjects and DMD patients. Furthermore, N- and C-terminal fragments of titin were most frequently detected among various identified titin-derived peptides^10^; they were detected not only in the urine of DMD patients but also in the urine of patients with other muscular dystrophies, such as Becker muscular dystrophy and limb-girdle muscular dystrophy^10^. Therefore, it has been suggested that N- and C-terminal titin fragments could be employed as molecular markers for the screening of many types of muscular dystrophies and also for the non-invasive monitoring of muscle disease progression, response to therapeutic treatment, and exercise-related muscle burden^10^. However, currently available western blot analysis using anti-titin antibodies is semi-quantitative and technically complex, as well as being insufficiently sensitive to measure the concentrations of titin fragments in urine throughout the disease stages in patients of all ages^10^. It may also be unsuitable to evaluate the outcome of therapeutic treatment(s) and to investigate exercise-related muscle damage and repair processes.

Therefore, in this study, we established a highly sensitive sandwich ELISA (Enzyme-Linked Immuno Sorbent Assay) system to measure the abundance of the titin N-terminal fragment in urine. We also examined whether the established ELISA can cover the whole range of biologically relevant concentrations of the titin N-terminal fragment in urine. Our results indicate that the developed ELISA system is sufficiently sensitive for this purpose. The results also suggest that it could be suitable for screening purposes and also for evaluation of disease-stage, outcomes of therapeutic treatment(s), and investigations of exercise-related muscle damage and repair processes.

## Results

### Preparation of immunogen incorporating human titin N-terminal peptide fragment

To prepare a suitable immunogen, a vector encoding a fusion protein consisting of glutathione S-transferase (GST) peptide, the N-terminal 200 residues of titin (Titin-N) and a His-tag was designed, and the recombinant protein (designated as GST-Titin-N-His) was synthesized in *E. coli*.

**Figure 1:**
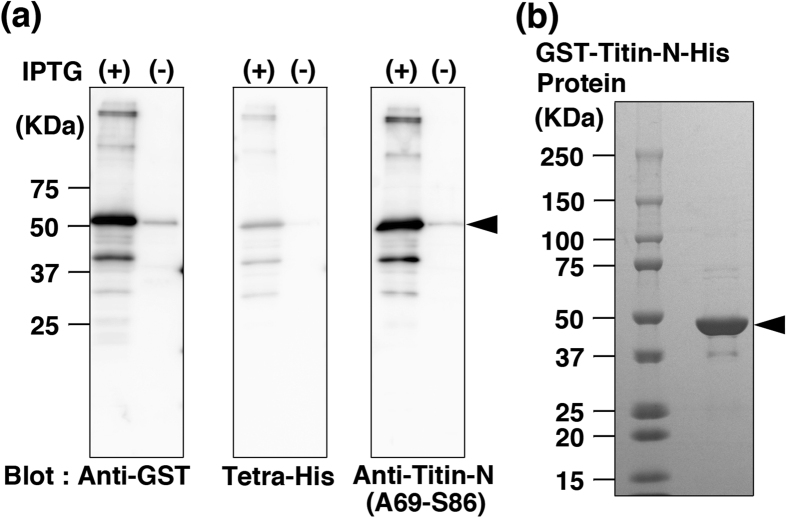
Preparation of human Titin-N fragment protein. (**a**) The GST- and Histidine-tagged protein of human Titin-N fragment (GST-Titin-N-His) produced in *E. coli* was detected as an approximately 50 kDa protein by anti-GST (left), anti-His (center), and anti-Titin-N (A69-S86) (right) antibodies. Original gel image was attached in supplementary information (Supplementary Figure 1). (**b**) A 50 kDa GST-Titin-N-His protein was purified using Ni-NTA agarose (Qiagen), analyzed by SDS-gel electrophoresis and stained. IPTG: Isopropyl β-D-1-thiogalactopyranoside, GST: glutathione S-transferase.

As shown in Fig. 1, GST-Titin-N-His was detected by antibodies against the GST-peptide, His-tag and human titin peptide (A69-S86) as a protein of approximately 50 kDa (Fig. 1a, arrowhead), which is consistent with the size calculated from the amino acid sequence. The recombinant protein was affinity-purified using Ni-NTA agarose (Fig. 1b).

### Characterization of antibodies raised against GST-Titin-N-His

**Figure 2:**
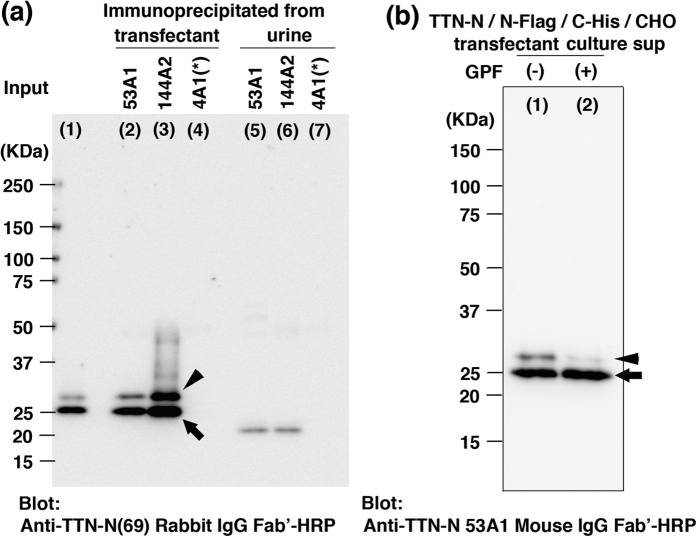
Characterization of antibodies, 53A1 and 144A2. (**a**) 0.5 mL of culture supernatant of recombinant CHO-K1 cells expressing FLAG-Titin-N-His and 3 mL of urine from a healthy volunteer were immuno-precipitated with 2 μg of 53A1, 144A2 and 4A1 antibodies. The Flag-Titin-N-His fusion proteins in the conditioned medium of CHO-K1 cells (lane 1), the Flag-Titin-N-His fusion proteins immuno-precipitated from the conditioned medium of CHO-K1 cells with 53A1 (lane 2), 144A2 (lane 3), and 4A1 (lane 4) and the titin proteins immuno-precipitated from urine of healthy volunteer with 53A1(lane 5), 144A2 (lane 6) and 4A1 (lane 7) were applied on SDS-PAGE and blotted with anti-Titin-N (A69-S86) rabbit IgG Fab’-HRP. 4A1 (Anti-Titin-C fragment mouse IgG monoclonal antibody), denoted by asterisk was used as a negative control. (**b**) Culture supernatant of recombinant CHO-K1 cells expressing FLAG-Titin-N-His proteins was incubated with glycopeptidase F (GPF, Takara Bio), and subjected to SDS-PAGE (Lane 2) and the gel was blotted with 53A1, an anti-Titin-N Mouse IgG Fab’-HRP.

Immuno-precipitation and western blotting analysis were performed to confirm whether the selected antibodies had the ability to react against the native titin N-fragment in aqueous conditions. The titin N-fragments in the urine of healthy volunteers and in the conditioned medium of CHO-K1 cells that expressed Flag-Titin N-His peptides were immuno-precipitated with 53A1 and 144A2 antibodies, and blotted with anti-Titin-N (A-69 to S-86) rabbit IgG Fab’-HRP. As shown in Fig. 2a, Flag-Titin-N-His peptides were detected as dual bands. This could be due to post-translational modification or an unexpected artifact. Although titin is a non-glycosylated cytoplasmic protein, the Flag-Titin-N-His peptide was synthesized as a secreted form, which contain two putative N-glycosylation sites. Therefore, we examined the effect of digestion with peptide N-glycosidase F (GPF, Takara Bio). The enzyme reaction resulted in a decrease in the intensity of the upper band (arrowhead) and an increase in the lower band (arrow), as shown in Fig. 2b. This suggests that the lower-molecular-weight band is due to the titin peptide, while the higher-molecular-weight band is due to glycosylated titin peptide. In addition, the native Titin-N fragment in urine was detected as a single band of approximately 21–22 kDa, and no other extra band was identified (Fig. 2a), suggesting that 53A1 and 144A2 antibodies are specific for the Titin N-fragment and available for the construction of a sandwich ELISA for the Titin-N fragment.

### Establishment of the quantitative ELISA system for the Titin-N fragment

**Figure 3:**
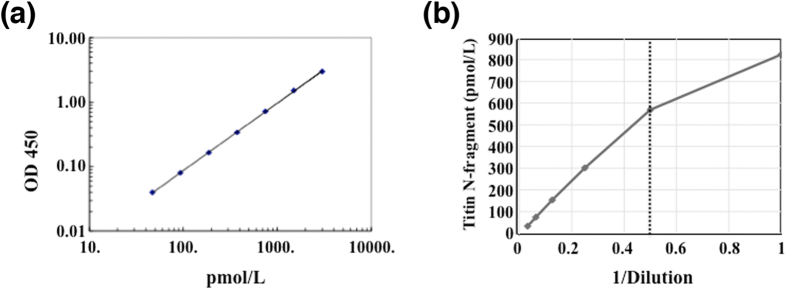
Characterization of ELISA for Titin-N fragment. (**a**) Standard curve for calculation of the Titin-N fragment. The linearity range for the Titin-N fragment is from 46.9 to 3000 (pmol/L). (**b**) The linearity of measured values for serial dilutions in human urine. Sample 1 is 2-fold-diluted urine, and thus a dilution ratio of 4-fold or more is suitable for urine samples.

We developed a sandwich ELISA system for the human Titin-N fragment in urine using a combination of the antibodies 53A1 and 144A2. Antibody 144A2 was used as the capture antibody and 53A1 was used as the detection antibody. The standard dose-response curve for the Titin-N fragment showed excellent linear (R^2^ = 1) when plotted on a log/log scale over a range of 46.9 to 3,000 pmol/L (Fig. 3a). Next, we validated the basic performance of the Titin-N fragment ELISA system. The intra- and inter-assay coefficients of variation (CV) were calculated by measuring solutions containing three spiked doses of Flag-Titin-N-His fusion-proteins. Fusion-proteins were added to PBS to prepare solutions of three doses that corresponded to the high (H), middle (M) and low (L) ranges of the standard curves (these solutions were termed as quality control (QC) sample H, M and L). Intra-assay precision was determined by 24 repeated measurements of each QC sample in a plate and exhibited the coefficients of variation (CVs) of 3.8% in H, 4.2% in M and 8.6% in L (Table 1). The inter-assay precision was determined by assessing each QC sample across seven different plates with quintuple wells. The obtained CVs were 5.7% in H, 5.7% in M and 7.7% in L (Table 2).

Moreover, a linearity test and a recovery test were performed to assess the influence of factors in the urine samples that may interfere with the reaction. The linearity test was performed to determine the suitable dilution ratio for the assay; high linearity was observed with a dilution ratio of 4-fold or more in human urine samples (Fig. 3b). As for the recovery test, samples containing different concentrations of GST-Titin-N-His peptides were measured and their recovery rates were validated in terms of the difference between the measured concentrations and the theoretical concentrations. The recovery rates were nearly 100% with a 5-fold dilution (Table 3).

Taken together, at least a 5-fold dilution was required to avoid interference, and the sensitivity of this assay kit was calculated to be 27.9 pmol/L using the guidelines provided by the National Committee for Clinical Laboratory Standards (NCCLS) Evaluation Protocols. Thus, the newly established ELISA system was evaluated as reliable from the standpoint of precision. In addition, two antibodies (53A1 and 144A2) used for the ELISA construction cross-reacted to the rat Titin-N fragment, but not to the mouse counterpart; thus, our ELISA kit would be useful for the analysis of physiological activities and pathological events in muscle of rat.

### Titin-N fragment concentrations in the urine of healthy volunteers

To examine the reference ranges for the Titin-N fragment in the urine of healthy volunteers, we next analyzed the levels of urinary Titin-N fragment under everyday life conditions. The concentrations of Titin-N fragment in the urine of 18 healthy adults are indicated in Table 4. Because urine volume and components are affected by physiological conditions such as changes in kidney function, water and food intake, dehydration, sweating, physical actions, life-style, age, sex and so on^11^,^12^, we decided to normalize the titin N-fragment concentrations by urinary creatinine (Cr) contents. Thus, data are shown as Titin-N/Cr-ratio (pmol/mg/dl) (Tables 4, 5, 6 and 7 and Supplementary Table 1–8).

Next, the circadian fluctuations of urinary Titin-N fragment were examined in 6 adult volunteers (58-, 39-, 38-, 70-, 42-, and 34-year-old males) (Table 5 and Supplementary Table 1–5). The circadian fluctuations of Titin-N fragment concentration in urine appear to lie within the range between approximately 0.5 nmol/L and 5 nmol/L. When Titin-N fragment concentrations were normalized to urinary creatinine contents, the circadian fluctuations of Titin-N levels (Titin-N/Cr-ratios) seemed to become smaller.

In addition to adult volunteers, the Titin-N fragment levels of growing-phase volunteers, a 2.5-year-old female and a 5-year-old male, were analyzed. As shown in Table 6 and Supplementary Table 6, the Titin-N fragment levels of children appeared similar to those of adults.

Overall, we examined the urinary Titin-N concentrations of 18 healthy adults (18 samples) and the circadian fluctuations of Titin-N levels in urine of 6 healthy adults (33 samples) and 2 children (15 samples). Although the sample sizes are quite limited, our results at least indicate the usefulness of the Titin-N ELISA system. Nevertheless, to establish the normal range of Titin-N in healthy volunteers, it will be necessary to perform a large-scale, comprehensive cohort study of healthy male and female volunteers with a wide rang of ages.

### The time course fluctuations of Titin-N levels in urine after exercise

**Figure 4:**
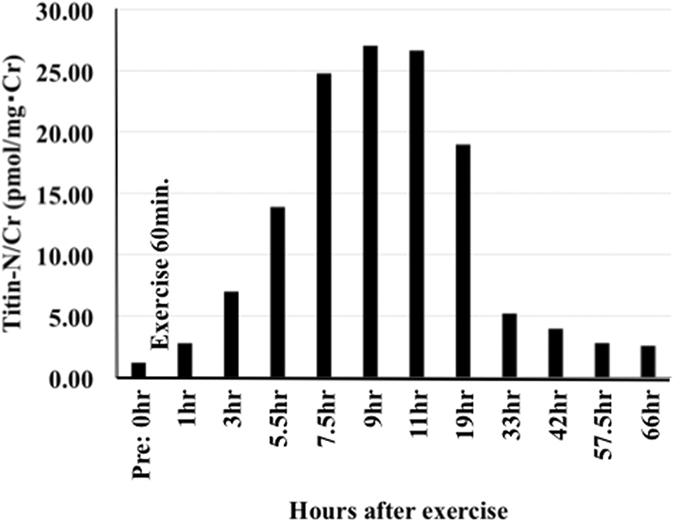
Time course of Titin-N fragment levels in the urine after exercise. The time course of Titin-N fragment levels in the urine after exercise is shown schematically. Detailed results are given in Supplementary Table 7.

Because it has been reported that eccentric muscle fiber contraction triggers cytoskeletal and sarcomeric disruption, including calpain-mediated degradation of titin^13^, we tested the effects of exercise. As expected, large increases in Titin-N fragment levels were observed in the urine of two volunteers (29-year-old male; 60.86 nmol/L, 23-year-old male; 460.71 nmol/L), collected just after running (32 km/8 hours). Since sequential processes of muscle damage, inflammatory response, hypertrophic response and sarcomerogenesis and fiber repair are induced after muscle damage^2^,^5^,^6^,^7^, we analyzed the fluctuations of Titin-N fragment levels after exercise (10 km running/1 hour, 39-year-old male). As shown in Fig. 4 and Supplementary Table 7, Titin-N/Cr-ratios gradually increased soon after exercise, reached to the highest levels at approximately 5 to 6 hours after exercise (and were maintained for approximately 12 hours), and then gradually decreased to everyday life levels. Similar profiles were observed with two more volunteers (Supplementary Tables 8 and 9). However, these data are insufficient to evaluate the effects of exercise, which are influenced by many factors, including the training status of the subject, intensity and duration of exercise, the degree and type of fatigue, age and sex^13^, and a well-organized, much larger-scale cohort study would be needed to address these issue.

### Titin-N fragment levels in DMD patients

In DMD, the loss of dystrophin induces instability of the sarcolemma, particularly during contraction and, consequently, leads to a massive Ca^2+^ influx into muscle fibers and the subsequent disturbance of calpain activity, resulting in the degradation of titin and sarcomere structure^2^,^5^,^6^. This fact suggests that titin fragments could be used for the non-invasive monitoring of muscle disease progression and the response to therapeutic treatment. However, the presently available western blot method for Titin-N and -C fragments is only applicable for samples from young ambulatory DMD patients, and is insufficiently sensitive to measure samples from older non-ambulatory DMD patients; thus, making it difficult to evaluate muscle disease progression and the response to therapeutic treatment^10^. To confirm whether our ELISA system is applicable to measure the Titin-N fragment in urine throughout the disease stages and at all patient ages, we tried to measure the Titin-N fragment in urine samples from non-ambulatory DMD patients. The Titin-N fragment levels in the urine of 18- and 20- year-old non-ambulatory DMD patients were 2.04 and 7.07 nmol/L, respectively (Table 7). It is important to note that the muscle mass of DMD patients decrease as disease progresses^14^ and thus the muscle mass of these two DMD patients, whose disease was advanced, was expected to be much smaller than those of healthy volunteers and young ambulatory DMD patients. We measured the creatinine levels to evaluate the muscle mass^14^,^15^, and as expected, the creatinine levels in urine of these two DMD patients were much lower than those of healthy volunteers. Consequently, although the urinary Titin-N concentrations of these two patients were similar to those of healthy volunteers, the Titin-N/Cr-ratios of both patients were much higher than those of healthy volunteers (Tables 4 and 7). These results suggested, to our surprise, that severe muscle injury was continuing even in relatively old non-ambulatory patients at an advanced stage of the disease. It is informative to compare the serum levels of creatine kinase-M (CK-M) with the Titin-N levels and Titin-N/Cr- ratios measured in this study, because CK-M was the first marker reported to be elevated in the serum of DMD patients^16^ and is commonly used as a blood-based biomarker for muscular dystrophy to evaluate the level of muscle damage and necrosis^17^,^18^,^19^. The serum CK-M levels of the 18- and 20- year-old non-ambulatory DMD patients were 3104U/L and 707U/L, respectively (Table 7). These results are very interesting, but to assess their significance and to establish whether or not the ELISA system for Titin-N fragment will be useful for pathological and clinical diagnosis of muscle dystrophy, it will be necessary to complete a large-scale, comprehensive cohort study of DMD patients between the age of disease on-set (around 3 years) and death (around 30 years), including measurements of not only urinary Titin-N concentrations, but also CK-M and other biomarkers for DMD, such as muscle specific miRNAs, as well as muscle biomarker proteins.

## Discussion

For the screening of muscular dystrophies, and also for monitoring disease progression and evaluating the efficacy of therapeutic approaches, as well as for investigating exercise-related sarcomeric disruption and repair processes, reliable and less invasive outcome measures are indispensable. For this purpose, several types of molecular biomarkers have been proposed. CK-M was first reported to be elevated in the serum of DMD patients^16^ and has been used for screening DMD patients at birth^18^,^19^. However, serum CK-M is sensitive to a number of factors, including exercise, age, race, and pharmacological interventions (e.g. statin use). Serum CK-M levels may decline in older patients with more advanced disease on account of reduced muscle mass and do not always correlate with other read-outs of muscle pathology^20^, thus they are of limited value for monitoring disease progression and evaluating the efficacy of therapeutic interventions. Other muscle proteins, namely carbonic anhydrase III (CA-III)^21^ and myoglobin^22^, were also identified to be elevated in the serum of DMD and Becker-type muscular dystrophy patients. In addition to these muscle-specific proteins, muscle inflammation-associated proteins, MMP9, TIMP1 and osteopontin are used as markers for DMD patients^23^.

Muscle-specific micro RNAs (miRNAs such as miR-1, miR-133a, miR-133b and miR-206, referred as dystromirs) leak into the bloodstream and are highly elevated in the serum of DMD patients and muscular dystrophy animal models^24^,^25^. Cacchiarelli *et al*.^24^ and Roberts *et al*.^26^ found that the elevated levels of miR-1 and miR-206 in serum of mdx mice were decreased almost to the levels of wild type mice by the restoration of dystrophin (exon skipping treatment). A subsequent cohort study of DMD patients^27^ found that circulating serum miR-1, miR-133a,b and miR-206 were significantly increased in DMD patients and the extent of their elevation was related to the disease severity, namely, blood levels of dystromirs were higher in patients with milder disease than in more severely affected patients. But, although dystrophin restoration by exon skipping treatment decreased the circulating dystromirs levels in DMD patients, this did not reach statistical significance, possibly due to the small number of patients enrolled and the short duration of exon skipping treatment. Nevertheless, Zaharieva *et al*.^27^ suggested that miR-1 and miR-133 might be useful as exploratory biomarkers for monitoring the progression of muscle weakness. Thus, dystromirs are candidates for less-invasive biomarkers to monitor disease progression and the outcome of therapeutic interventions.

However, in the context of the current clinical biochemical laboratory environment, protein-based biomarkers (i.e. ELISA) are perhaps preferable. Along this line, Rouillon *et al*.^28^ found that two fragments of the myofibrillar structural protein myomesin-3 (MYOM3) were abnormally present in sera of DMD patients, limb-girdle muscular dystrophy type 2D (LGMD2D) and animal models of these diseases. Importantly, levels of MYOM3 fragments were restored toward wild-type levels when dystrophin expression was restored by exon skipping in mdx mice and when α-sarcoglycan (SGCA) expression was induced in SGCA deficient mice, suggesting that the MYOM3 fragments is a potential therapy-responsive protein biomarker for DMD and other neuromuscular disorders. In addition, Coenen-Stass *et al*.^29^ profiled 1,129 proteins in the serum of wild-type and mdx mice by utilizing an aptamer-based proteomics approach and identified multiple novel, therapy-responsive protein biomarkers in the serum of the mdx mice with potential utility in DMD patients.

Regarding biomarkers that are able to evaluate the health-related aspects of muscle damages, which can be modulated by regular physical activity and exercise, various biomarkers and combinations of several different types of approaches have been proposed^30^. However, there is no gold standard for monitoring most of the processes, as the values of biomarkers for health-related physical activities and exercise-initiated muscle burden are dependent on many factors, such as the training status of the subject, the intensity and duration of exercise, the degree and type of fatigue, as well as age and sex^31^.

Currently, the establishment of highly sensitive and accurate outcome measure is becoming an increasingly important issue, as increasing numbers of drug development programs focusing on slowing or preventing progressive muscle pathogenesis are being established^32^,^33^,^34^,^35^,^36^,^37^,^38^. Thus, the establishment of less-invasive and technically uncomplicated biomarker(s) to monitor the disease-stage and the outcome of therapeutic treatment(s) is urgently needed. There is also an unmet need to establish biomarkers to investigate exercise-related muscle damage and repair processes^30^,^31^,^39^,^40^. To resolve these situations, in this study, we developed an ELISA to measure titin N-terminal fragment in human urine, and we demonstrated that the established ELISA is highly sensitive and available for the detection of a wide range of concentrations of titin N-terminal fragment in urine. The sensitivity of our ELISA was calculated to be 27.9 pmol/L (lower limit of ELISA) and at least a 5-fold dilution is recommended to avoid the influence of interfering reactions. Therefore, the actual lower limit of the ELISA was calculated to be 139.5 pmol/L, indicating that the titin N-fragment levels (~nmol/L order) present in the urine from non-ambulatory DMD patients and healthy volunteers under everyday-life conditions were much higher than the actual lower limit of the ELISA. In addition, we confirmed that the linearity of the ELISA is good and sufficient to measure a wide range of concentrations of titin N-fragment, from normal levels to very high levels. Therefore, the ELISA should be applicable to measure the titin N-terminal fragment in urine samples from older non-ambulatory DMD patients and healthy volunteers under everyday life conditions, as well as in urine samples from younger ambulatory DMD patients and athletes undergoing eccentric exercise. This suggests that the ELISA for the Titin-N terminal fragment established in this study could be a useful tool for the screening of muscular dystrophies and also for monitoring the progression of muscle disease, evaluating the efficacy of therapeutic approaches, and investigating exercise-related sarcomeric disruption and repair processes.

## Materials and Methods

### Preparation of recombinant human titin N-fragment proteins

A human heart cDNA library was generated using a first-strand cDNA synthesis kit (GE Healthcare) from human heart total RNA (Clonetech). The cDNA encoding the human titin N-fragment, 1–200 amino acids (Titin-N), was amplified by PCR, using forward (5′-AACTCGAGAGGTACTTCTTCTTCACCTTA-3′) and reverse (5′-AAGGATCCATGACAACTCAAGCACCGACG-3′) primers; each primer contains the restriction sites for XhoI and BamHI. Following PCR amplification, double-stranded cDNA was digested with XhoI and BamHI and inserted into the pGEX6P-1 vector in which the glutathione S-transferase (GST) sequence and Histidine-tag (His) were located at N and C terminal regions of multi-cloning sites, respectively. The digested double-stranded cDNA was also inserted into the pcDNA3.1(+) vector that was embedded with signal peptides and a Flag-tag (Flag) at the N-terminus and a His-tag at the C-terminus. Subsequently, the pGEX6P-1 construct was introduced into BL21 competent cells (DE3) (Thermo Fisher Scientific) and a single colony that contained a recombinant plasmid was isolated. Then, the transformed colony was cultured in LB medium containing 50 μg/mL ampicillin, and protein expression was induced by IPTG (Isopropyl β-D-1-thiogalactopyranoside) for 24 hours. The cultured *E. coli* was harvested and a recombinant fusion protein of GST peptide, Titin-N (1–200) and His-tag (designated as GST-Titin-N-His) was purified using Ni-NTA agarose (Qiagen). The pcDNA3.1(+) construct was transfected into Chinese hamster ovary cells (CHO-K1) using lipofectamine 2000 (Thermo Fisher Scientific). Cells stably expressing a fusion protein of signal-sequence, Flag-tag, human Titin-N(1–200) and His-tag (Flag-Titin-N-His) were selected by 1 mg/ml G418 sulfate (Thermo Fisher Scientific) and cloned by limiting dilution. The stable transformant cells were cultured in TIL media (Immuno-biological Laboratories) containing 10% FCS. The Flag-Titin-N-His fusion protein was purified from the culture supernatant using Ni-NTA agarose. Western blot analyses revealed that the molecular weights of GST-Titin-N-His and Flag-Titin-N-His proteins were consistent with those calculated from their amino acid sequences, suggesting that the two human titin-N fusion proteins were faithfully synthesized in *E. coli* and CHO-K1 cells. The purities of the isolated GST-Titin-N-His proteins were estimated from the staining patterns of SDS-gel electrophoresis, and their concentration was evaluated by comparison with the band intensities of serial dilutions of co-electrophoresed bovine serum albumin, using a densitometer (Multi gauge; GE Healthcare). Flag and Titin-N fusion protein (Flag-Titin-N-His) synthesized in CHO-K1 cells was used as a standard protein. The concentration of Flag-Titin-N-His was calculated by comparing the concentrations of GST-Titin-N-His (the concentration measured by an ELISA kit for GST was used as the reference) and Flag-Titin-N-His measured by the ELISA kit for Titin N-fragment (established in this study).

### Subjects

The human studies were conducted in accordance with the principles of the Declaration of Helsinki “Ethical Principles for Medical Research Involving Human Subjects” in Kobe Gakuin University, in the Graduate School of Medicine, Kobe University and in Immuno-biological Laboratories Co., Ltd. The study protocol was approved by the ethics committees on human research at the Graduate School of Medicine, Kobe University (Approval number: 1709 for the preparation and analysis of urine samples from DMD patients), at the Faculty of Rehabilitation, Kobe Gakuin University (Approval number; 5 for the preparation and analysis of urine samples from healthy volunteers) and at Immuno-biological Laboratories Co., Ltd (Approval number; 030 for the analysis of human urine samples). Since no invasive procedure was performed and no individual clinical and genetic data were collected, this research dose not meet the criteria for biomedical research, as defined by the human study guidelines in Japan. Written informed consent was obtained from subjects and from the mothers of the two children prior to enrollment. Urine samples from healthy volunteers and non-ambulatory DMD patients were stored at −80 °C immediately after collection and thawed just before analysis.

### Animals

Animal experimentation was conducted in accordance with the Japanese guidelines for the protection of vertebrate animals used for experimental purposes. All animal care and experimental procedures were approved by the Animal Care and Experimentation Committee of Immuno-biological Laboratories Co.,Ltd. (Approval number; 201505C for the production of mouse monoclonal antibodies against titin-N fragment). All mice were purchased from Charles River Laboratories Japan, Inc. and were maintained in SPF conditions in the animal facility on a 12-hour light-dark cycle, with water and standard rodent diet (MF; Oriental Yeast Co.,Ltd.) *ad libitum* according to the institutional guidelines of Immuno-biological Laboratories Co., Ltd. Six-week-old female BDF1 mice were injected subcutaneously with 50 μg of the recombinant Titin-N fragment (GST-Titin-N-His) in complete Freund’s adjuvant. After three additional injections of immunogen and a final booster injection (see Preparation of anti-human Titin-N fragment antibodies), mice were sacrificed by cervical dislocation and lymphocytes were prepared.

### Preparation of anti-human titin N-fragment antibodies

First, we prepared rabbit polyclonal antibodies. Rabbits were immunized with the synthetic peptides EVSWFRDGQVISTSTL-(C) and (C)-AVTKANSGRYSLKATNGS, which were coupled with bovine thyroglobulin as a carrier protein. These peptide sequences correspond to the regions of Glutamate-36 (E-36) to Leucine-51 (L-51) and Alanine-69 (A-69) to Serine-86 (S-86) of the human titin protein, respectively. Cysteine residues at the C-terminal and N-terminal ends of the two peptides were attached for the coupling reaction with carrier protein. The immunoglobulin (Ig)G fractions against these two peptide sequences were identified from the serum of immunized rabbits using Thiol-Sepharose-embedded affinity-columns (GE Healthcare), to which either of two antigen-peptides was coupled. Secondly, we established mouse monoclonal antibodies. Six-week-old female BDF1 mice were injected subcutaneously with 50 μg of the recombinant titin-N fragment (GST-Titin-N-His) in complete Freund’s adjuvant. Three additional injections of immunogen in incomplete Freund’s adjuvant were given every other week. Ten days after the final injection, the mice were boosted with 20 μg of immunogen, and lymphocytes from the immunized mice were fused with the myeloma cell line X63-Ag8.653. Hybridoma cells were selected in hypoxanthine/aminopterin/thymidine medium. Reactive clones against the antigen were selected by evaluating the sensitivities of sandwich ELISAs that were constructed by the combination of the rabbit polyclonal antibody against synthetic peptide (E-36 to L51), and the selected candidate monoclonal antibodies were used as the capture antibody and the detection antibody, respectively. Several positive clones were selected by the limiting dilution method and two clones (53A1 and 144A2) were finally selected for ELISA construction.

### Immunoprecipitation and western blotting analysis

The reactivities of antibodies were assessed by immuno-precipitation and western blotting methods. 0.5 mL of culture supernatant of recombinant CHO-K1 cells expressing FLAG-titin-N-His and 3 mL of urine from a healthy volunteer were incubated with 2 μg of 53A1, 144A2 and 4A1 (anti-titin-C fragment mouse IgG monoclonal antibody) antibodies at 4 °C for 2 hours, respectively. Then, the equilibrated Protein G Sepharose (GE Healthcare Japan) was added and allowed to react at 4 °C with rotation. Two hours later, the immuno-complex with Protein G Sepharose was washed with PBST, collected by centrifugation, and re-suspended in 30 μL of 2x loading buffer (a solution containing 2% SDS, 10% glycerol, 50 mM Tris-HCl (pH 6.8) and 100 mM 2-mercaptoethanol). After heat treatment at 95 °C for 3 min, the supernatant was applied to 10% SDS-gel electrophoresis and detected by horseradish peroxidase (HRP)-conjugated anti-Titin N(A69-S86) rabbit IgG Fab’.

Signals were detected with a cooled CCD camera (LAS-3000mini, Fuji-firm. Lens type: VRF43LMD, Filter: 1 Through, Image reader: LAS-3000IR ver2.2W).

### Establishment of the ELISA system for quantification of human Titin N-fragment

We developed a sandwich Titin N-Fragment ELISA that is a combination of two selected antibodies, 53A1 and 144A2. Antibody 144A2 was used as a capture antibody and HRP-conjugated 53A1 Fab’ was used as a detection antibody. Microtiter plates (96 wells) were coated by being filled with 100 μl/well of 100 mM carbonate buffer (pH 9.5) containing 0.5 μg/well of 144A2 overnight at 4 °C. Then the plates were washed with PBS and blocked with 200 μL/well of 1% (w/v) bovine serum albumin (BSA) in PBS containing 0.05% NaN_3_ overnight at 4 °C. After two washes with PBST, test samples and recombinant human Titin N-fragment peptide (Flag-Titin-N-His, a standard that was serially diluted with 1% BSA in PBST) were added into the wells of the coated microtiter plates in duplicate, and incubated at 37 °C for 1 hour. After four washes with PBST, 100 μl of HRP-conjugated 53A1 mouse IgG Fab’ was added to each well and incubated at 37 °C for 30 minutes. The wells were washed with PBST five times, and then 100 μl of freshly prepared tetramethyl benzidine solution was added to each well as a substrate. The plates were incubated in the dark for 30 minutes at room temperature. The reaction was terminated by adding 100 μL of 1 N H_2_SO. The absorbance of the solution was measured at 450 nm with an ELISA reader (E-Max; Molecular Devices, Sunnyvale, CA, USA).

To assess the intra- and inter-assay precision of the ELISA, the intra- and inter-assay coefficients of variation (CVs) were calculated by measuring three different doses with recombinant human Titin N-fragment peptide (Flag-Titin-N-His, 1–200 a.a.) (designated as quality control (QC) samples of the high (H), middle (M) and low (L) ranges of the standard curves). Intra-assay variability was determined by 24 repeated measurements of three different doses of samples in a plate. Inter-assay precision was determined by assessing three different doses of samples across seven different plates with quintuple wells. Moreover, a linearity test and a recovery test were performed for assessing the influence of factors in the urine samples that may interfere with the reaction. To evaluate the linearity of the assay, serial dilutions of Flag-Titin-N-His peptide (1–200 a.a.) were measured in human urine samples. The accuracy of recovery assay in the presence of human urine was determined by serially diluting Flag-Titin-N-His peptides (1–200 a.a.) in human urine, and the recovery rate was validated in term of the difference between the measured concentration and the theoretical concentration. The sensitivity for this kit was determined based on the guidelines provided by the National Committee for Clinical Laboratory Standards (NCCLS) Evaluation Protocols. (National Committee for Clinical Laboratory Standards Evaluation Protocols, SC1, (1989) Villanova, PA: NCCLS).

## Additional Information

**How to cite this article:** Maruyama, N. *et al*. Establishment of a highly sensitive sandwich ELISA for the N-terminal fragment of titin in urine. *Sci. Rep.* **6**, 39375; doi: 10.1038/srep39375 (2016).

**Publisher's note:** Springer Nature remains neutral with regard to jurisdictional claims in published maps and institutional affiliations.

## Supplementary Material

Supplementary Information

## Figures and Tables

**Table 1:** Intra-Assay Precision.

QC	Measured Values (pmol/L)	SD	CV (%)	n
H	1189.1	44.97	3.8	24
M	358.6	15.23	4.2	24
L	102.8	8.85	8.6	24

The intra-assay results were derived from analysis of 24 replicates of QC sample. CV (%) is calculated from SD Value/Measured Value. SD; Standard deviation. CV: Coefficient of variation.

**Table 2:** Inter-Assay Precision.

QC	Measured Values (pmol/L)	SD	CV (%)	n
H	1188.21	68.02	5.7	7
M	364.99	20.79	5.7	7
L	107.61	8.27	7.7	7

The inter-assay results were based on 7 separate measurements of each QC in quintuplicate. CV (%) is calculated from SD Value/Measured Value. SD; Standard deviation. CV: Coefficient of variation.

**Table 3:** Recovery rates of titin N-fragment in human urine.

Sample	Added Flag-titin-N (pmol/L)	Theoretical Value (pmol/L)	Measured Values (pmol/L)	%
Human Urine (1291.5 pmol/L)	1500.0	2791.5	2823.6	101.5
	375.0	1666.5	1717.7	103.1
	46.9	1338.4	1330.2	99.4

Purified titin N-fragment proteins (FLAG-Titin-N-CHO, 1500, 375, 46.9 pmol/L each) were added to human urine samples (5-fold diluted) in which 1291.5 pmol/L of titin N-fragment was included. The recovery rates (%) at high, middle and low concentrations were calculated from the measured values (the mean value of duplicate samples) against the theoretical values, respectively. The recovery rates in all concentrations were nearly100% in human urine.

**Table 4:** Titin-N fragment levels in urine of healthy adult volunteers.

Sample (age, sex)	Values	Result (pmlo/L)	Mean result (pmol/L)	Std. Dev.	CV (%)	Dilution	Calculated Titin-N (nmol/L)	Cr (mg/dl)	Titin-N/Cr (pmol/mg/dl)
1 (39, M)	0.274	282.4	289.9	10.48	3.6	5	1.45	121.29	1.20
	0.289	297.3							
2 (58, M)	0.673	744.3	765.1	29.42	3.8	5	3.83	148.21	2.58
	0.712	785.9							
3 (39, M)	0.344	389.2	391.4	3.09	0.8	5	1.96	165.65	1.18
	0.348	393.6							
4 (28, M)	0.344	455.7	468.7	18.38	3.9	5	2.34	103.21	2.27
	0.365	481.7							
5 (50, M)	0.247	334.2	345.0	15.20	4.4	5	1.72	156.21	1.10
	0.264	355.7							
6 (38, M)	0.706	728.2	730.7	3.564	0.5	5	3.65	333.84	1.09
	0.711	733.2							
7 (70, M)	0.348	364.6	359.0	7.975	2.2	5	1.80	49.56	3.62
	0.337	353.4							
8 (42, M)	0.156	226.6	222.4	5.937	2.7	5	1.11	74.16	1.50
	0.150	218.2							
9 (34, M)	0.981	1004.3	995.3	12.737	1.3	5	4.98	229.45	2.17
	0.963	986.3							
10 (42, M)	0.471	490.2	482.1	11.519	2.4	5	2.41	195.84	1.23
	0.455	474.0							
11 (46, M)	1.020	1043.3	1039.3	5.655	0.5	5	5.20	201.00	2.59
	1.012	1035.3							
12 (47, M)	0.580	600.9	594.3	9.312	1.6	5	2.97	148.90	2.00
	0.567	587.7							
13 (37, M)	0.771	793.7	792.2	2.134	0.3	5	3.96	189.72	2.09
	0.768	790.6							
14 (44, M)	0.391	408.7	406.6	2.891	0.7	5	2.03	80.13	2.54
	0.387	404.6							
15 (59, M)	2.035	2048.9	2052.8	5.564	0.3	5	10.26	144.77	7.09
	2.043	2056.7							
16 (46, F)	0.115	123.3	120.1	4.465	3.7	5	0.60	30.49	1.97
	0.109	117.0							
17 (37, F)	0.131	140.1	142.7	3.706	2.6	5	0.71	46.35	1.54
	0.136	145.3							
18 (35, F)	0.539	559.3	552.7	9.329	1.7	5	2.76	124.13	2.23
	0.526	546.1							

The concentrations of creatinine in urine samples were measured by the creatinase-sarcosine oxidase-POD method (Special Reference Laboratory Co., Tokyo, Japan). The value of Titin N- fragment concentration was corrected by the value of creatinine and is shown as creatinine ratio (pmol/mg · Cr) = Titin N-fragment(nmol/L) ÷ Cr(mg/dl) × 100.

**Table 5:** The circadian fluctuations of Titin-N fragment concentrations in urine of healthy volunteer 1 (58-year-old male).

Sample (Age, Sex, Time)	Values	Result (pmol/L)	Mean result (pmol/L)	Std. Dev.	CV (%)	Dilution	Calculated Titin-N (nmol/L)	Cr (mg/dl)	Titin-N/Cr (pmol/ mg/dl)
1 (58, M) 7:00	0.673	744.3	765.1	29.420	3.8	5	3.83	148.21	2.58
	0.712	785.9							
1 (58, M) 10:00	0.808	887.9	896.4	12.000	1.3	5	4.48	147.23	3.04
	0.824	904.9							
1 (58, M) 11:30	0.594	659.7	661.9	3.033	0.5	5	3.31	101.50	3.26
	0.598	664.0							
1 (58, M) 15:30	0.957	1045.6	1039.8	8.206	0.8	5	5.20	199.39	2.61
	0.946	1034.0							
1 (58, M) 21:00	0.645	714.3	702.1	17.404	2.5	5	3.51	108.08	3.25
	0.622	689.7							
1 (58, M) 21:30	0.160	185.7	184.5	1.589	0.9	5	0.92	28.08	3.29
	0.158	183.4							

**Table 6:** The circadian changes of Titin-N fragment concentrations in urine of young volunteer 1 (2.5-year-old female).

Sample	Values	Result (pmol/L)	Mean result (pmol/L)	Std. Dev.	CV (%)	Dilution	Calculated Titin-N (nmol/L)	Cr (mg/dl)	Titin-N/Cr (pmol/mg/dl)
Day 1 8:00	0.235	319.0	311.4	10.806	3.5	5	1.56	92.12	1.69
	0.223	303.7							
Day 1 12:00	0.157	218.6	221.2	3.689	1.7	5	1.11	34.11	3.24
	0.161	223.8							
Day 1 20:00	0.149	208.1	212.1	5.550	2.6	5	1.06	44.32	2.39
	0.155	216.0							
Day 2 8:00	0.220	299.9	308.8	12.614	4.1	5	1.54	104.30	1.48
	0.234	317.7							
Day 2 12:00	0.321	427.2	428.4	1.761	0.4	5	2.14	80.67	2.66
	0.323	429.7							
Day 2 20:00	0.252	340.6	344.4	5.365	1.6	5	1.72	24.13	7.14
	0.258	348.1							
Day 3 8:00	0.227	308.8	344.2	50.078	14.5	5	1.72	116.77	1.47
	0.283	379.6							
Day 3 12:00	0.183	252.4	256.9	6.389	2.5	5	1.28	40.15	3.20
	0.190	261.4							

**Table 7:** Titin-N fragment concentrations in urine samples of DMD patients.

Patient (Age, Sex)	Values	Result (pmol/L)	Mean result (pmol/L)	Std. Dev.	CV (%)	Dilution	Calculated Titin-N (nmol/L)	Cr (mg/dl)	Titin-N/Cr (pmol/mg/dl)	CK-M (U/L)
1 (18, M)	0.312	415.9	408.4	10.602	2.6	5	2.04	1.53	133.48	3104
	0.300	400.9								
2 (20, M)	1.108	1361.0	1414.2	75.321	5.3	5	7.07	2.72	259.97	707
	1.201	1467.5								
