# Supplementary material for: Establishment of a highly sensitive sandwich ELISA for the N-terminal fragment of titin in urine
Source: Sci Rep. 2016 Dec 19;6:39375. doi: 10.1038/srep39375 (PMC5171804; doi:10.1038/srep39375)
Supplement: Supplementary Information [file srep39375-s1.doc]

**Supplementary Information (SREP-16-30494)**

**Establishment of a highly sensitive sandwich ELISA for the N-terminal fragment of titin in urine**

Nobuhiro Maruyama^1^, Tsuyoshi Asai^2^, Chiaki Abe^3^, Akari Inada^3^, Takeshi Kawauchi^3^, Kazuya Miyashita^1^, Masahiro Maeda^1^, Masafumi Matsuo^2^, Yo-ichi Nabeshima^3^*

1. Diagnostic & Research Reagents Division, Immuno-Biological Laboratories Co., Ltd.

1091-1 Naka, Fujioka-shi, Gunma 375-0005, JAPAN

2. Department of Physical Therapy, Faculty of Rehabilitation, Kobe Gakuin University

1-1-3 Minatojima-Minamimachi Chuo-ku, Kobe 650-0047 Japan

3. Laboratory of Molecular Life Science, Institute of Biomedical Research and Innovation Foundation for Biomedical Research and Innovation

2-2 Minatojima- Minamimachi Chuo-ku, Kobe 650-0047 Japan

*Correspondence: Laboratory of Molecular Life Science, Institute of Biomedical Research and Innovation Foundation for Biomedical Research and Innovation,

2-2 Minatojima-Minamimachi Chuo-ku, Kobe 65-0047 Japan.

TEL 81-78-303-3681 E-mail: nabemr@lmls-kobe.org

***Keywords*:** Muscle biomarker, Titin, Urine, Muscle damage, ELISA, Duchenne muscular dystrophy, Exercise

**Supplementary Figure 1**

**Original gel image of Figure 1 (a)**

**Left panel Center panel Right panel**

**
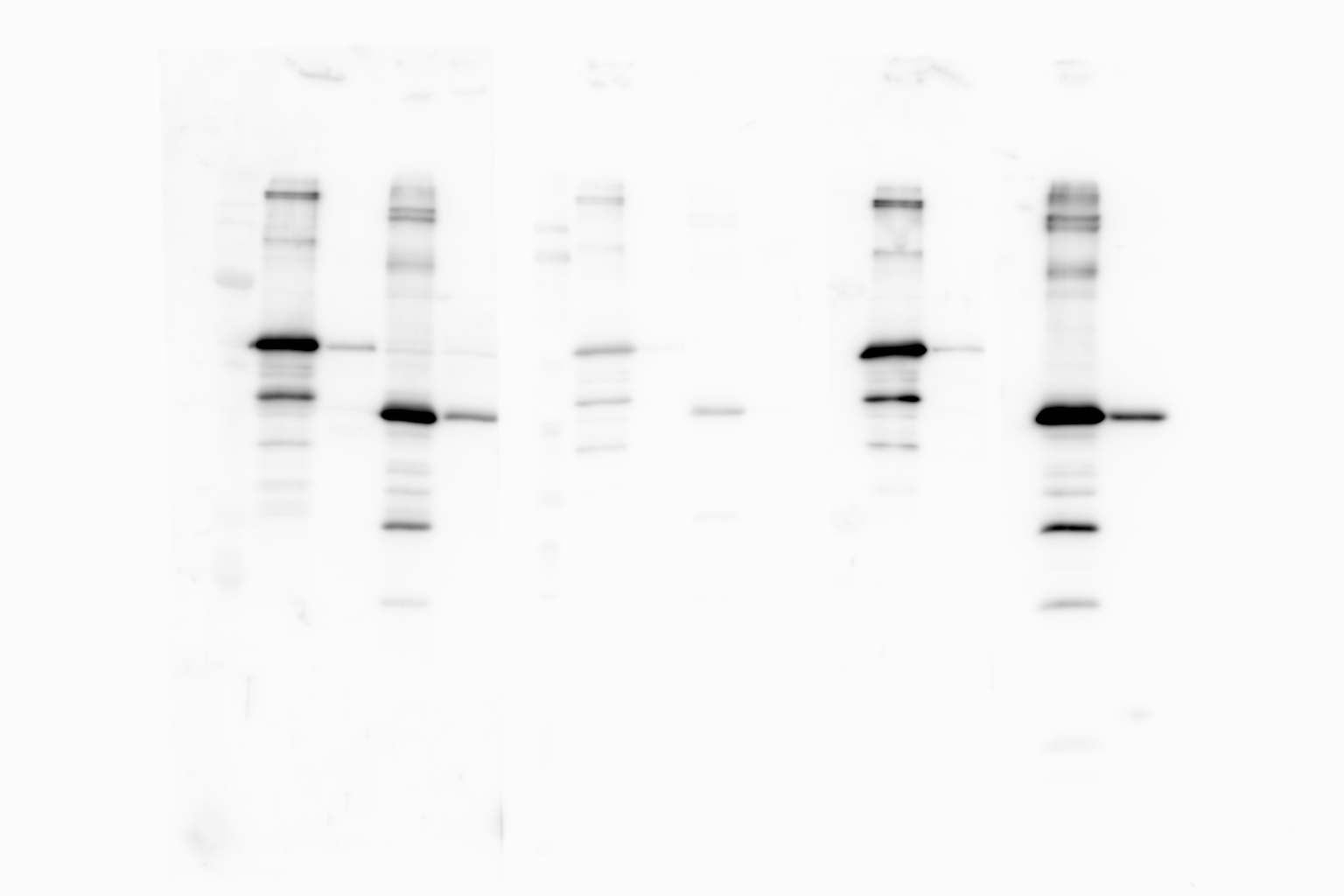
**

**Supplementary Table 1 The circadian fluctuations of titin-N fragment concentrations in urine of healthy volunteer 2 (39-year-old male)**

| **Sample**  **(Age, Sex) Time** | **Values** | **Result**  **(pmol/L)** | **Mean**  **result**  **(pmol/L)** | **Std. Dev.** | **CV**  **(%)** | **Dilution** | **Calculated**  **Titin-N**  **(nmol/L)** | **Cr**  **(mg/dl)** | **Titin-N/Cr**  **(pmol/**  **mg/dl)** |
| --- | --- | --- | --- | --- | --- | --- | --- | --- | --- |
| **2 (39, M)**  **6:30** | **0.344** | **389.2** | **391.4** | **3.092** | **0.8** | **5** | **1.96** | **165.65** | **1.18** |
|  | **0.348** | **393.6** |  |  |  |  |  |  |  |
| **2 (39, M)**  **8:45** | **0.092** | **108.6** | **113.2** | **6.469** | **5.7** | **5** | **0.57** | **41.59** | **1.37** |
|  | **0.100** | **117.8** |  |  |  |  |  |  |  |
| **2 (39, M)**  **11:00** | **0.166** | **192.4** | **185.1** | **10.326** | **5.6** | **5** | **0.93** | **66.64** | **1.40** |
|  | **0.153** | **177.8** |  |  |  |  |  |  |  |
| **2 (39, M)**  **16:00** | **0.249** | **284.8** | **280.9** | **5.476** | **1.9** | **5** | **1.40** | **159.98** | **0.90** |
|  | **0.242** | **277.0** |  |  |  |  |  |  |  |
| **2 (39, M)**  **24:00** | **0.419** | **470.9** | **435.5** | **50.049** | **11.05** | **5** | **2.18** | **177.75** | **1.23** |
|  | **0.354** | **400.1** |  |  |  |  |  |  |  |

**The concentrations of creatinine in urine samples were measured by the creatinase-sarcosine**

**oxidase-POD method (Special Reference Laboratory Co., Tokyo, Japan). The value of Titin N- fragment concentration was corrected by the value of creatinine and is shown as creatinine ratio (pmol/mg・Cr)= Titin N-fragment(nmol/L)÷Cr(mg/dl) x 100**

**Supplementary Table 2 The circadian fluctuations of titin-N fragment concentrations in urine of healthy volunteer 3 (38-year-old male)**

| **Sample**  **(Age, Sex) Time** | **Values** | **Result**  **(pmol/L)** | **Mean**  **result**  **(pmol/L)** | **Std. Dev.** | **CV**  **(%)** | **Dilution** | **Calculated**  **Titin-N**  **(nmol/L)** | **Cr**  **(mg/dl)** | **Titin-N/Cr**  **(pmol/**  **mg/dl)** |
| --- | --- | --- | --- | --- | --- | --- | --- | --- | --- |
| **3 (38,M)**  **8:30** | **0.706** | **728.2** | **730.7** | **3.564** | **0.5** | **5** | **3.65** | **333.84** | **1.09** |
|  | **0.711** | **733.2** |  |  |  |  |  |  |  |
| **3 (38,M)**  **11:00** | **0.903** | **926.2** | **903.2** | **32.625** | **3.6** | **5** | **4.52** | **386.25** | **1.17** |
|  | **0.857** | **880.1** |  |  |  |  |  |  |  |
| **3 (38,M)**  **15:00** | **0.300** | **315.4** | **313.3** | **2.909** | **0.9** | **5** | **1.57** | **97.42** | **1.61** |
|  | **0.296** | **311.2** |  |  |  |  |  |  |  |
| **3 (38,M)**  **21:30** | **0.387** | **404.6** | **402.0** | **3.615** | **0.9** | **5** | **2.01** | **126.71** | **1.59** |
|  | **0.382** | **399.5** |  |  |  |  |  |  |  |
| **3 (38,M)**  **0:30** | **0.578** | **598.9** | **593.8** | **7.164** | **1.2** | **5** | **2.97** | **155.55** | **1.91** |
|  | **0.568** | **588.7** |  |  |  |  |  |  |  |

**Supplementary Table 3 The circadian fluctuations of titin-N fragment concentrations in urine of healthy volunteer 4 (70-year-old male)**

| **Sample**  **(Age, Sex) Time** | **Values** | **Result**  **(pmol/L)** | **Mean**  **result**  **(pmol/L)** | **Std. Dev.** | **CV**  **(%)** | **Dilution** | **Calculated**  **Titin-N**  **(nmol/L)** | **Cr**  **(mg/dl)** | **Titin-N/Cr**  **(pmol/**  **mg/dl)** |
| --- | --- | --- | --- | --- | --- | --- | --- | --- | --- |
| **4 (70, M)**  **6:00** | **0.348** | **364.6** | **359.0** | **7.975** | **2.2** | **5** | **1.80** | **49.56** | **3.62** |
|  | **0.337** | **353.4** |  |  |  |  |  |  |  |
| **4 (70, M)**  **8:00** | **0.288** | **303.0** | **297.9** | **7.282** | **2.4** | **5** | **1.49** | **58.19** | **2.56** |
|  | **0.278** | **292.7** |  |  |  |  |  |  |  |
| **4 (70, M)**  **12:00** | **0.306** | **321.5** | **312.8** | **12.365** | **4.0** | **5** | **1.56** | **72.84** | **2.15** |
|  | **0.289** | **304.0** |  |  |  |  |  |  |  |
| **4 (70, M)**  **14:00** | **0.177** | **188.1** | **186.1** | **2.946** | **1.6** | **5** | **0.93** | **52.13** | **1.78** |
|  | **0.173** | **184.0** |  |  |  |  |  |  |  |
| **4 (70, M)**  **18:00** | **0.231** | **244.2** | **239.5** | **6.588** | **2.8** | **5** | **1.20** | **66.00** | **1.81** |
|  | **0.222** | **234.9** |  |  |  |  |  |  |  |
| **4 (70, M)**  **20:30** | **0.267** | **281.4** | **275.2** | **8.755** | **3.2** | **5** | **1.38** | **76.24** | **1.80** |
|  | **0.255** | **269.0** |  |  |  |  |  |  |  |
| **4 (70, M)**  **23:30** | **0.336** | **352.3** | **343.6** | **12.338** | **3.6** | **5** | **1.72** | **75.52** | **2.27** |
|  | **0.319** | **334.9** |  |  |  |  |  |  |  |

**Supplementary Table 4 The circadian fluctuations of titin-N fragment concentrations in urine of healthy volunteer 5 (42-year-old male)**

| **Sample**  **(Age, Sex) Time** | **Values** | **Result**  **(pmol/L)** | **Mean**  **result**  **(pmol/L)** | **Std. Dev.** | **CV**  **(%)** | **Dilution** | **Calculated**  **Titin-N**  **(nmol/L)** | **Cr**  **(mg/dl)** | **Titin-N/Cr**  **(pmol/**  **mg/dl)** |
| --- | --- | --- | --- | --- | --- | --- | --- | --- | --- |
| **5 (42, M)**  **8:30** | **0.156** | **226.6** | **222.4** | **5.937** | **2.7** | **5** | **1.11** | **74.16** | **1.50** |
|  | **0.150** | **218.2** |  |  |  |  |  |  |  |
| **5 (42, M)**  **14:00** | **0.221** | **316.9** | **312.7** | **5.859** | **1.9** | **5** | **1.56** | **83.84** | **1.87** |
|  | **0.215** | **308.6** |  |  |  |  |  |  |  |
| **5 (42, M)**  **16:00** | **0.202** | **290.6** | **276.7** | **19.624** | **7.1** | **5** | **1.38** | **79.25** | **1.75** |
|  | **0.182** | **262.9** |  |  |  |  |  |  |  |
| **5 (42, M)**  **22:00** | **0.256** | **365.1** | **363.7** | **1.942** | **0.5** | **5** | **1.82** | **143.83** | **1.26** |
|  | **0.254** | **362.3** |  |  |  |  |  |  |  |
| **5 (42, M)**  **25:00** | **0.092** | **136.3** | **139.2** | **4.031** | **2.9** | **5** | **0.70** | **50.82** | **1.37** |
|  | **0.096** | **142.0** |  |  |  |  |  |  |  |

**Supplementary Table 5 The circadian fluctuations of titin-N fragment concentrations in urine of healthy volunteer 6 (34-year-old male)**

| **Sample**  **(Age, Sex)**  **Time** | **Values** | **Result**  **(pmol/L)** | **Mean**  **result**  **(pmol/L)** | **Std. Dev.** | **CV**  **(%)** | **Dilution** | **Calculated**  **Titin-N**  **(nmol/L)** | **Cr**  **(mg/dl)** | **Titin-N/Cr**  **(pmol/**  **mg/dl)** |
| --- | --- | --- | --- | --- | --- | --- | --- | --- | --- |
| **6 (34, M)**  **9:00** | **0.981** | **1004.3** | **995.3** | **12.737** | **1.3** | **5** | **4.98** | **229.45** | **2.17** |
|  | **0.963** | **986.3** |  |  |  |  |  |  |  |
| **6 (34, M)**  **11:00** | **0.478** | **497.4** | **492.3** | **7.196** | **1.5** | **5** | **2.46** | **59.51** | **4.14** |
|  | **0.468** | **487.2** |  |  |  |  |  |  |  |
| **6 (34, M)**  **14:00** | **0.822** | **844.9** | **827.3** | **24.875** | **3.0** | **5** | **4.14** | **157.68** | **2.62** |
|  | **0.787** | **809.8** |  |  |  |  |  |  |  |
| **6 (34, M)**  **20:00** | **0.073** | **78.9** | **77.8** | **1.504** | **1.9** | **5** | **0.39** | **10.56** | **3.69** |
|  | **0.071** | **76.8** |  |  |  |  |  |  |  |
| **6 (34, M)**  **25:00** | **0.191** | **275.4** | **273.3** | **2.945** | **1.1** | **5** | **1.37** | **36.29** | **3.77** |
|  | **0.188** | **271.2** |  |  |  |  |  |  |  |

**Supplementary Table 6 The circadian fluctuations of titin-N fragment concentrations in urine of young volunteer 2 (5-year-old male**)

| **Sample**  **(Age, Sex)**  **Time** | **Values** | **Result**  **(pmol/L)** | **Mean result**  **(pmol/L)** | **Std. Dev.** | **CV**  **(%)** | **Dilution** | **Calculated**  **Titin-N**  **(nmol/L)** | **Cr**  **(mg/dl)** | **Titin-N/Cr**  **(pmol/mg/dl)** |
| --- | --- | --- | --- | --- | --- | --- | --- | --- | --- |
| **2 (5, M)**  **6:40** | **0.110** | **161.9** | **152.0** | **14.060** | **9.3** | **5** | **0.76** | **40.74** | **1.86** |
|  | **0.096** | **142.0** |  |  |  |  |  |  |  |
| **2 (5, M)**  **10:00** | **0.199** | **286.5** | **280.2** | **8.827** | **3.1** | **5** | **1.40** | **61.46** | **2.28** |
|  | **0.190** | **274.0** |  |  |  |  |  |  |  |
| **2 (5, M)**  **10.30** | **0.193** | **278.1** | **288.5** | **14.694** | **5.1** | **5** | **1.44** | **61.85** | **2.33** |
|  | **0.208** | **298.9** |  |  |  |  |  |  |  |
| **2 (5, M)**  **13:50** | **0.128** | **187.3** | **178.1** | **12.976** | **7.3** | **5** | **0.89** | **41.49** | **2.15** |
|  | **0.115** | **169.0** |  |  |  |  |  |  |  |
| **2 (5, M)**  **16:00** | **0.073** | **109.1** | **108.4** | **1.018** | **0.9** | **5** | **0.54** | **22.82** | **2.37** |
|  | **0.072** | **107.7** |  |  |  |  |  |  |  |
| **2 (5, M)**  **19:00** | **0.298** | **422.6** | **403.4** | **27.075** | **6.7** | **5** | **2.02** | **98.73** | **2.04** |
|  | **0.270** | **384.3** |  |  |  |  |  |  |  |
| **2 (5, M)**  **19:30** | **0.325** | **459.4** | **456.6** | **3.849** | **0.8** | **5** | **2.28** | **75.71** | **3.02** |
|  | **0.321** | **453.9** |  |  |  |  |  |  |  |

**Supplementary Table 7 The time course of Titin-N levels in urine after exercise**

**(38-year-old male, 10km running/60 min.)**

| **Sample** | **Values** | **Result**  **(pmol/L)** | **Mean result**  **(pmol/L)** | **Std. Dev.** | **CV**  **(%)** | **Dilution** | **Calculated**  **Titin-N**  **(nmol/L)** | **Cr**  **(mg/dl)** | **Titin-N/Cr**  **(pmol/mg/dl)** |
| --- | --- | --- | --- | --- | --- | --- | --- | --- | --- |
| **Pre**  **0 hr** | **0.274** | **282.4** | **289.9** | **10.48** | **3.6** | **5** | **1.45** | **121.29** | **1.20** |
|  | **0.289** | **297.3** |  |  |  |  |  |  |  |
| **Exercise 10km running/60 min.** | | | | | | | | | |
| **1 hrs** | **0.407** | **412.9** | **412.9** | **0** | **0** | **5** | **2.06** | **74.29** | **2.77** |
|  | **0.407** | **412.9** |  |  |  |  |  |  |  |
| **3 hrs** | **1.403** | **1354.3** | **1362.2** | **11.14** | **0.8** | **5** | **6.81** | **97.47** | **6.99** |
|  | **1.420** | **1370.1** |  |  |  |  |  |  |  |
| **5.5 hrs** | **2.265** | **2144.7** | **2158.8** | **19.92** | **0.9** | **5** | **10.79** | **77.69** | **13.89** |
|  | **2.296** | **2172.9** |  |  |  |  |  |  |  |
| **7.5 hrs** | **1.117** | **1008.2** | **1095.2** | **9.92** | **0.9** | **50** | **54.76** | **220.99** | **24.78** |
|  | **1.132** | **1102.2** |  |  |  |  |  |  |  |
| **9 hrs** | **1.055** | **1030.1** | **1022.2** | **11.27** | **1.1** | **50** | **51.11** | **189.10** | **27.03** |
|  | **1.038** | **1014.2** |  |  |  |  |  |  |  |
| **11 hrs** | **1.129** | **1099.4** | **1096.6** | **3.97** | **0.4** | **50** | **54.83** | **205.84** | **26.64** |
|  | **1.123** | **1093.8** |  |  |  |  |  |  |  |
| **19 hrs** | **0.775** | **766.2** | **766.2** | **0** | **0** | **50** | **38.31** | **201.97** | **19.00** |
|  | **0.775** | **766.2** |  |  |  |  |  |  |  |
| **33 hrs** | **1.362** | **1316.3** | **1314.9** | **1.97** | **0.1** | **5** | **6.57** | **126.75** | **5.18** |
|  | **1.359** | **1313.5** |  |  |  |  |  |  |  |
| **42 hrs** | **0.575** | **575.3** | **572.0** | **4.76** | **0.8** | **5** | **2.86** | **72.29** | **3.96** |
|  | **0.568** | **568.6** |  |  |  |  |  |  |  |
| **57.5 hrs** | **0.327** | **334.7** | **332.2** | **0.7** | **0.2** | **5** | **1.67** | **59.88** | **2.89** |
|  | **0.326** | **333.7** |  |  |  |  |  |  |  |
| **66 hrs** | **0.658** | **654.8** | **653.4** | **2.03** | **0.3** | **5** | **3.27** | **127.07** | **2.77** |
|  | **0.655** | **651.9** |  |  |  |  |  |  |  |

**Supplementary Table 8 The time course of Titin-N levels in urine after exercise**

**(39-year-old (A), male, 5 km running/30 min.)**

| **Sample** | **Values** | **Result**  **(pmol/L)** | **Mean result**  **(pmol/L)** | **Std. Dev.** | **CV**  **(%)** | **Dilution** | **Calculated**  **Titin-N**  **(nmol/L)** | **Cr**  **(mg/dl)** | **Titin-N/Cr**  **(pmol/mg/dl)** |
| --- | --- | --- | --- | --- | --- | --- | --- | --- | --- |
| **Pre,**  **0 hr** | **0.239** | **263.6** | **264.2** | **0.772** | **0.3** | **5** | **1.32** | **104.76** | **1.26** |
|  | **0.240** | **264.7** |  |  |  |  |  |  |  |
| **Exercise 30 min** | | | | | | | | | |
| **3 hrs** | **0.526** | **575.4** | **581.3** | **8.417** | **1.4** | **5** | **2.91** | **93.71** | **3.10** |
|  | **0.537** | **587.3** |  |  |  |  |  |  |  |
| **8.5 hrs** | **0.587** | **641.4** | **633.3** | **11.468** | **1.8** | **50** | **31.66** | **137.59** | **23.01** |
|  | **0.572** | **625.1** |  |  |  |  |  |  |  |
| **17 hrs** | **0.507** | **554.8** | **565.6** | **15.309** | **2.7** | **50** | **28.28** | **134.46** | **21.03** |
|  | **0.527** | **576.5** |  |  |  |  |  |  |  |
| **32.5 hrs** | **0.497** | **544.0** | **548.9** | **6.891** | **1.3** | **5** | **2.72** | **190.53** | **1.44** |
|  | **0.506** | **553.7** |  |  |  |  |  |  |  |

**Supplementary Table 9 The time course of Titin-N levels in urine after exercise**

**(39 years old (B), male 16km running/100min.)**

| **Sample** | **Values** | **Result**  **(pmol/L)** | **Mean result**  **(pmol/L)** | **Std. Dev.** | **CV**  **(%)** | **Dilution** | **Calculated**  **Titin-N**  **(nmol/L)** | **Cr**  **(mg/dl)** | **Titin-N/Cr**  **(pmol/mg/dl)** |
| --- | --- | --- | --- | --- | --- | --- | --- | --- | --- |
| **Pre**  **0 hr** | **0.496** | **515.7** | **516.2** | **0.719** | **0.1** | **5** | **2.58** | **85.22** | **3.03** |
|  | **0.497** | **516.7** |  |  |  |  |  |  |  |
| **Exercise 100 min.** | | | | | | | | | |
| **2.5 hrs** | **1.099** | **1192.8** | **1177.3** | **22.024** | **1.9** | **5** | **5.89** | **18.04** | **3.27** |
|  | **1.070** | **1161.7** |  |  |  |  |  |  |  |
| **8.5 hrs** | **2.129** | **2294.6** | **2306.9** | **17.342** | **0.8** | **5** | **11.53** | **87.98** | **13.11** |
|  | **2.152** | **2319.2** |  |  |  |  |  |  |  |
| **16.5 hrs** | **2.244** | **2417.2** | **2411.4** | **8.290** | **0.3** | **5** | **12.06** | **141.48** | **8.25** |
|  | **2.233** | **2405.5** |  |  |  |  |  |  |  |
| **32.5 hrs** | **1.347** | **1458.9** | **1449.7** | **12.882** | **0.9** | **5** | **7.25** | **64.05** | **11.32** |
|  | **1.330** | **1440.6** |  |  |  |  |  |  |  |
